
JOURNAL INFORMATION
==============================
NLM Title Abbreviation: Inter Econ
Journal ID: Inter Econ
NLM Journal ID: 101086399

Inter Economics

ISSN: 0020-5346

ARTICLE INFORMATION
==============================
PMCID: PMC8853015
DOI: 10.1007/s10272-022-1018-9
Article ID: 1018
Article version: 1
Subjects: Forum

Redesigning EU Fiscal Rules After COVID-19

Electronic publication date: 2022 Feb 12
Print publication date: 2022
Volume: 57
Issue: 1
First page: 4
Last page: 4
Copyright: © The Author(s) 2022
License: Open Access: This article is distributed under the terms of the Creative Commons Attribution 4.0 International License (https://creativecommons.org/licenses/by/4.0/).
Open Access funding provided by ZBW — Leibniz Information Centre for Economics.
License URL: https://creativecommons.org/licenses/by/4.0/

issue-copyright-statement: © The Author(s) 2022

==============================

DOI: 10.1007/s10272-022-1018-9

### Redesigning EU Fiscal Rules After COVID-19

**The policy responses to the COVID-19 pandemic have highlighted two related issues in the EU fiscal surveillance framework in the current low interest rate regime: the increasing distance of some member states from the threshold for government debt of 60% of GDP as well as the lack of clarity about the future of the EU fiscal framework once the suspension of the general escape clause comes to an end. The current framework might not sufficiently take into account the need for government investment to drive the green and digital transition. All these elements point to a new debate about EU fiscal rules. This Forum enhances the discussion on the realisation of a credible fiscal framework that takes into account the current macroeconomic context. The following contributions, based on a recent conference jointly organised by *Intereconomics* and the Centre for European Policy Studies, provide a critical assessment of the current state of the debate on European fiscal rules, examine the recent European Fiscal Board's reform proposal for the EU fiscal framework, draw on the experience of the national independent fiscal institutions and consider the idea of a social golden rule for public investments.**

### Economic Recovery in the Age of COVID-19

**Carlos Cuerpo**, Tesoro and International Financing, Madrid, Spain.

### EU Fiscal Rules: A Look Back and the Way Forward

**Klaus Regling**, European Stability Mechanism, Luxembourg, Luxembourg.

### The Economics of Fiscal Rules and Debt Sustainability

**Roel Beetsma**, University of Amsterdam, Netherlands; and European Fiscal Board, Brussels, Belgium.

### Towards New Fiscal Rules in the Euro Area?

**Catherine Mathieu**, OFCE, Paris, France.
**Henri Sterdyniak**, OFCE, Paris, France.

### EU Fiscal Governance Reforms: A Perspective of Independent Fiscal Institutions

**Sebastian Barnes**, Network of EU Independent Fiscal Institutions, Brussels, Belgium; Irish Fiscal Advisory Council, Dublin, Ireland.

### A Golden Rule for Social Investments: How to Do It

**Cinzia Alcidi**, Centre for European Policy Studies, Brussels, Belgium.
**Francesco Corti**, Centre for European Policy Studies, Brussels, Belgium; and University of Milan, Italy.
**Daniel Gros**, Centre for European Policy Studies, Brussels, Belgium.
